# Immunotherapy utilization in stage IIIA melanoma: less may be more

**DOI:** 10.3389/fonc.2024.1336441

**Published:** 2024-02-06

**Authors:** Alexander E. Frey, Daniel M. Kerekes, Sajid A. Khan, Thuy T. Tran, Harriet M. Kluger, James E. Clune, Stephan Ariyan, Mario Sznol, Jeffrey J. Ishizuka, Kelly L. Olino

**Affiliations:** ^1^ Department of Surgery, Yale University School of Medicine, New Haven, CT, United States; ^2^ Department of Medicine (Medical Oncology), Yale University School of Medicine, New Haven, CT, United States

**Keywords:** melanoma, immunotherapy, skin cancer, guidelines, treatment

## Abstract

**Background:**

Immunotherapy agents are approved for adjuvant treatment of stage III melanoma; however, evidence for survival benefit in early stage III disease is lacking. Current guidelines for adjuvant immunotherapy utilization in stage IIIA rely on clinician judgment, creating an opportunity for significant variation in prescribing patterns. This study aimed to characterize current immunotherapy practice variations and to compare patient outcomes for different prescribing practices in stage IIIA melanoma.

**Study design:**

Patients with melanoma diagnosed from 2015-2019 that met American Joint Committee on Cancer 8th edition criteria for stage IIIA and underwent resection were identified in the National Cancer Database. Multiple imputation by chained equations replaced missing values. Factors associated with receipt of adjuvant immunotherapy were identified. Multivariable Cox proportional hazards regression compared overall survival across groups.

**Results:**

Of 4,432 patients included in the study, 34% received adjuvant immunotherapy. Patients had lower risk-adjusted odds of receiving immunotherapy if they were treated at an academic center (OR=0.48, 95%CI=0.33-0.72, p<0.001 vs. community facility) or at a high-volume center (OR=0.69, 0.56-0.84, p<0.001 vs. low-volume). Immunotherapy receipt was not associated with risk-adjusted survival (p=0.095). Moreover, patients treated at high-volume centers experienced longer overall risk-adjusted survival than those treated at low-volume centers (HR=0.52, 0.29-0.93, p=0.030). Risk-adjusted survival trended toward being longer at academic centers than at community centers, but the difference was not statistically significant.

**Conclusion:**

Academic and high-volume centers utilize significantly less adjuvant immunotherapy in stage IIIA melanoma than community and low-volume centers without compromise in overall survival. These findings suggest that this population may benefit from more judicious immunotherapy utilization.

## Introduction

Advances in immunotherapy have dramatically changed the management and outcomes of stage III melanoma (1). Major milestones in the treatment of stage III melanoma include Federal Drug Administration (FDA) approval of the anti-CTLA-4 monoclonal antibody ipilimumab in 2015 and approval of the anti-PD-1 monoclonal antibodies nivolumab and pembrolizumab in 2017 and 2019, respectively (2–4). However, stage III melanoma, as defined by the American Joint Committee on Cancer (AJCC) 8^th^ edition, represents a wide range of disease and outcomes: patients with stage IIIA disease have a 5-year melanoma-specific survival (MSS) of 93%, compared to 32% for stage IIID patients (5). And while the evidence for benefit of immunotherapy agents in the more lethal late stage III melanoma is more robust, the evidence for benefit in the comparably survivable stage IIIA is inconclusive.

To date, completed randomized trials investigating immunotherapy in stage III melanoma have either excluded IIIA patients altogether or have failed to show significant benefit in this subpopulation. The CheckMate-238 trial that compared adjuvant nivolumab to adjuvant ipilimumab in melanoma did not include patients with stage IIIA disease (6). On subgroup analysis, the KEYNOTE-054 trial that compared adjuvant pembrolizumab to placebo for patients with stage III melanoma did not show a statistically significant difference in distant metastasis-free survival for stage IIIA patients, unlike for stage IIIB and IIIC patients (7). Finally, the EORTC-18071 trial that compared adjuvant ipilimumab to placebo in stage III melanoma patients failed to find a statistically significant difference in risk of recurrence or death for the stage IIIA subgroup (8). Moreover, all of these studies analyzed stage IIIA patients classified by AJCC 7^th^ edition criteria, a subgroup with a significantly lower MSS than stage IIIA patients classified by 8^th^ edition criteria (9).

This lack of clear and convincing evidence that stage IIIA melanoma patients benefit from adjuvant immunotherapy has led to guidelines that rely on clinician judgment and shared decision-making. The current National Comprehensive Cancer Network (NCCN) guidelines for treatment of cutaneous melanoma state that “In patients with very-low-risk stage IIIA disease (non-ulcerated primary ≤2 mm thickness, sentinel lymph node metastasis diameter <1 mm), the toxicity of adjuvant therapy may outweigh the benefit.” (10) Additionally, the current American Society of Clinical Oncology (ASCO) and European Society for Medical Oncology (ESMO) guidelines for treatment of cutaneous melanoma recommend consideration of adjuvant immunotherapy in stage IIIA patients only if they have at least 1 mm of involved lymph node (11, 12).

Moreover, any potential benefit of immunotherapy must be balanced with the associated costs and risks. A typical course of treatment with pembrolizumab for one year can cost in excess of $150,000 for the drug alone – not including costs associated with drug administration, travel, and lost wages (13, 14). For those who can afford it, 77-99% will experience a treatment-related side effect (12), sometimes severe enough to require hospitalization or discontinuation of treatment (15). This complicated risk-benefit analysis, compounded by disparities in provider comfort-level and accessibility to immunotherapy agents, has led to facility-level variation in use of immunotherapy even in metastatic melanoma (16, 17), in which evidence of benefit is clear and consistent (7, 18, 19).

Given the unclear survival benefit of adjuvant immunotherapy for patients with stage IIIA melanoma and the ambiguity of current management guidelines, we hypothesized that adjuvant immunotherapy utilization in stage IIIA melanoma would vary significantly between facilities. This study aimed to characterize recent practice patterns in the use of immunotherapy after resection for stage IIIA melanoma across different facility types and volumes and to compare patient outcomes in relation to any variations in immunotherapy practice.

## Methods

### Database and study population

The National Cancer Database (NCDB) is a clinical oncology database that reports demographic variables, treatment details, and outcomes for all patients with a cancer diagnosis treated at a Commission on Cancer-accredited facility (20). The NCDB Participant User Files for melanoma were acquired for patients diagnosed between 2015 and 2019. Only patients 18 years or older with stage IIIA disease as defined by the 8^th^ edition AJCC Staging Manual were included (21). For cases diagnosed in 2018 and 2019, 8^th^ edition AJCC stage was a variable reported by the NCDB. Cases diagnosed between 2015 and 2017 were reported by the NCDB with a 7^th^ edition AJCC stage, and were restaged with 8^th^ edition criteria using reported ulceration status, Breslow depth, and 7^th^ edition AJCC nodal stage. Cases with discordance between Breslow depth and pathologic T stage (for example, Breslow depth 0.5 mm, stage pT2) were excluded, as were patients with unknown immunotherapy status and those who received neoadjuvant immunotherapy. Only those who received surgical treatment of the primary tumor were included.

All patients who received immunotherapy received it in the adjuvant setting. The NCDB classifies therapeutic agents according to the Surveillance, Epidemiology, and End Results Program (SEER) Antineoplastic Drugs Database (22). For example, targeted therapies such as vemurafenib are categorized as chemotherapy and anti-PD1 agents such as nivolumab and pembrolizumab are categorized as immunotherapy along with oncolytic viruses such as talimogene laherparepvec. Patients that are treated with an agent as part of a clinical trial are captured by the NCDB.

As the NCDB is a deidentified database, this research study did not qualify as human subjects research and did not meet criteria for review by the Institutional Review Board.

### Statistical analysis

The primary outcome was receipt of adjuvant immunotherapy. The primary predictors were facility type and facility volume. Facility volume was classified on the basis of the number of patients with stage IIIA melanoma treated at each facility over the time frame of the study: low (≤50th percentile, 1-2 patients treated), intermediate (>50th to ≤75th percentile, 3-6 patients treated), and high (>75th percentile, ≥7 patients treated) volume facilities were defined.

Demographic and clinical characteristics were reported for the study group, stratified by immunotherapy status. Differences in characteristics between the groups were assessed using chi-squared (χ2) tests and independent samples two-tailed t-tests. Temporal trends in rate of immunotherapy administration were examined, stratified by a variety of patient demographic and clinical variables. Kaplan-Meier survival analysis was used to calculate survival, and log-rank testing compared unadjusted overall survival of patients based on immunotherapy status and facility characteristics. Patients lost to follow up were censored at time lost. Listwise deletion was used for this preliminary analysis.

Multiple imputation with chained equations was then used to impute missing values for patient race (n=27 [0.6%]), ethnicity (n=82 [1.9%]), facility location (n=752 [17.0%]), facility urbanicity (n=122, [2.8%]), zip code median income (n=729 [16.4%]), insurance status (n=54 [1.2%]), facility type (n=752 [17.0%]), ulceration status (n=32 [0.7%]), and extent of lymph node surgery (n=53 [1.2%]) (23). Five imputed datasets were generated using the R package MICE (24).

Multivariable logistic regressions using the imputed datasets were performed to identify predictors of immunotherapy receipt. Covariates included in the analysis were age group (≤50, 51-70, >70), sex, race, ethnicity, facility location, facility urbanicity, zip code median income, insurance status, facility type, Charlson-Deyo comorbidity index, T-stage, N-stage, and ulceration and mitotic rate (0-1, 2-3, ≥4) of the primary tumor. Multivariable Cox hazards regressions adjusted for age group, sex, facility location, zip code income, facility type, Charlson-Deyo comorbidity index, T-stage, N-stage, ulceration status, mitotic rate, and scope of lymph node surgery were performed in the cohort as a whole in order to assess for an association of immunotherapy status and facility type with overall survival. The same hazard regressions were also performed in the immunotherapy and no immunotherapy subpopulations in order to estimate hazard ratios for overall survival associated with different patient and tumor factors. Of note, melanoma-specific survival is not reported by the NCDB.

Age was categorized into three groups for ease of interpretation and because these split points have been previously shown to be associated with timely access to melanoma treatment (25). Patients were categorized into mitotic rate groups of 0-1, 2-3, and ≥4 mitoses/mm^2^, as these split points have been previously demonstrated to hold the highest prognostic value for T1 and T2 melanomas (26).

Hospital volume was also an independent variable of interest; therefore, the multiple imputation and all associated analyses were repeated with volume status used in place of facility type. Hospital volume is a proxy for facility type and allowed for verification of the facility type results with another commonly used metric. Facility type and hospital volume were not included as covariates together in any analysis given the *a priori* concern for multicollinearity, as hospital volume is a defining criterion for facility type classification (27).

Data were analyzed using IBM SPSS Statistics, version 28 (IBM Corporation, Armonk, New York) and RStudio, version 1.4.1717 (R Foundation for Statistical Computing, Vienna, Austria). The analysis performed was exploratory in nature, therefore no measures were taken to reduce the inflated risk of type 1 error due to multiple comparisons, and p<0.05 was considered significant for all tests.

## Results

### Patient demographics

**Figure 1:**
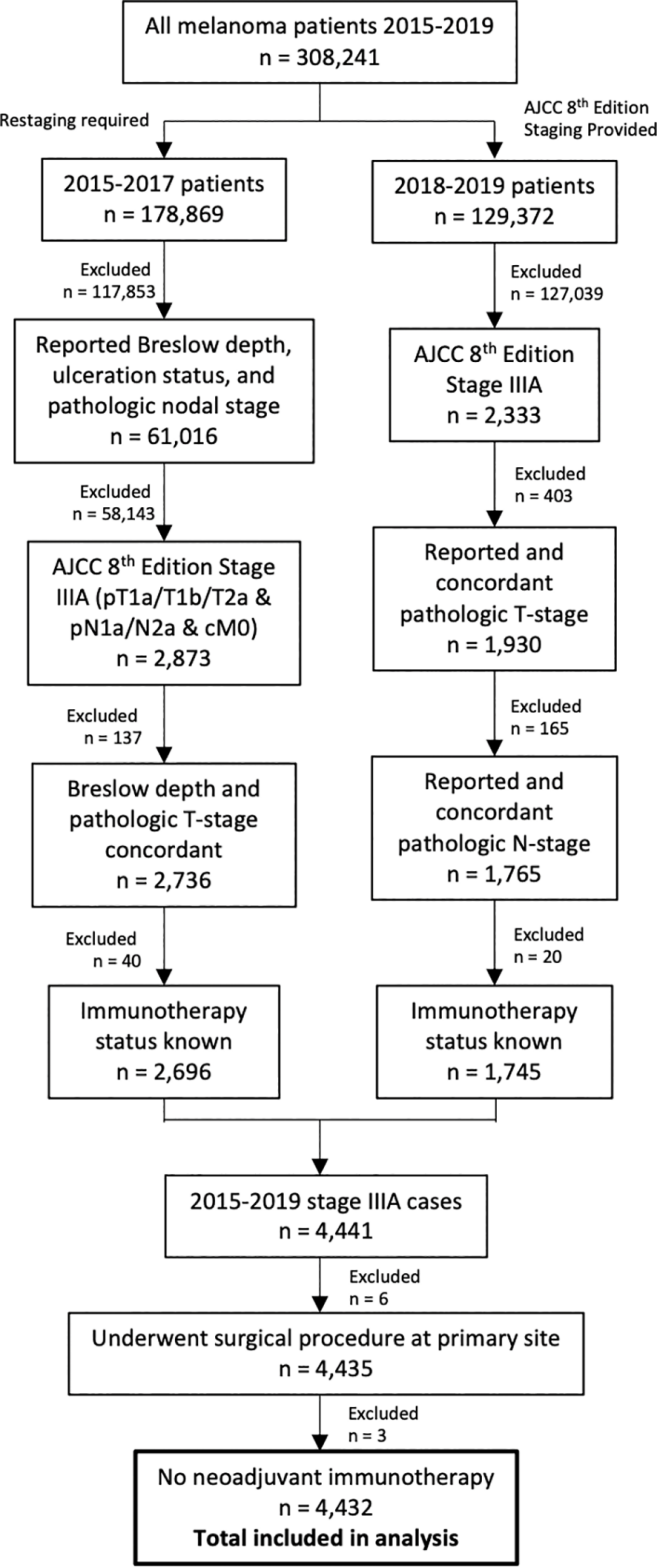
Flowchart of patient selection process. Patients diagnosed between 2015 and 2017 required restaging to American Joint Committee on Cancer 8^th^ Edition staging for inclusion.

There were 308,241 patients with melanoma diagnosed between 2015 and 2019. Of these, 4,432 patients had resected stage IIIA disease (**Figure 1**). Adjuvant immunotherapy was administered in 34% of cases. Compared to patients that did not receive immunotherapy, patients that received immunotherapy were significantly younger (mean age 53.0 vs. 56.9, p<0.001) and more often privately insured (69.2% vs. 58.6%, p<0.001) and treated at more than one facility (14.8% vs. 10.2%, p<0.001). From a disease burden standpoint, those that received immunotherapy were more likely to be nodal stage 2a (2-3 clinically occult nodes) rather than 1a (1 clinically occult node) (25.1% vs. 13.8%, p<0.001) (**Table 1**).

**Table 1:** Patient and facility characteristics stratified by immunotherapy receipt.

	No Immunotherapy	Received Immunotherapy	P-value
n (%)	2,923 (66.0)	1,509 (34.0)	–
Age, mean ± SD	56.9 ± 15.4	53.0 ± 14.7	<0.001
Sex, n (%)			
Male	1,543 (52.8)	810 (53.7)	0.574
Female	1,380 (47.2)	699 (46.3)	
Race, n (%)*			
White	2,870 (98.8)	1,479 (98.6)	0.450
Black	7 (0.2)	7 (0.5)	
Other	28 (1.0)	14 (0.9)	
Unknown	18 (0.6)	9 (0.6)	
Ethnicity, n (%)*			
Hispanic	53 (1.9)	37 (2.5)	0.167
Not Hispanic	2,806 (98.1)	1,454 (97.5)	
Unknown	64 (2.2)	18 (1.2)	
Facility Location, n (%)*			
Northeast	474 (19.0)	190 (16.0)	0.093
South	772 (31.0)	395 (33.2)	
Midwest	758 (30.4)	355 (29.8)	
West	486 (19.5)	250 (21.0)	
Unknown	433 (14.8)	319 (21.1)	
Facility County, n (%)*			
Metropolitan	2,376 (83.7)	1,248 (84.8)	0.613
Urban	425 (15.0)	204 (13.9)	
Rural	37 (1.3)	20 (1.4)	
Unknown	85 (2.9)	37 (2.5)	
Zip code median income, n (%)*			
< $38,000	243 (9.9)	117 (9.4)	0.673
$38,000 – $47,999	489 (19.9)	238 (19.2)	
$48,000 – $62,999	687 (27.9)	370 (29.8)	
≥$63,000	1,043 (42.4)	516 (41.6)	
Unknown	461 (15.8)	268 (17.8)	
Insurance, n (%)*			
None	45 (1.5)	29 (1.9)	<0.001
Private	1,714 (58.6)	1,044 (69.2)	
Medicaid	122 (4.2)	67 (4.4)	
Medicare	976 (33.4)	327 (21.7)	
Other government	25 (0.9)	29 (1.9)	
Unknown	41 (1.4)	13 (0.9)	
Facility Type, n (%)*			
Community	59 (2.4)	49 (4.1)	<0.001
Comprehensive	615 (24.7)	333 (28.0)	
Academic	1,318 (52.9)	530 (44.5)	
Network	498 (20.0)	278 (23.4)	
Unknown	433 (14.8)	319 (21.1)	
Volume, n (%)			
Low	300 (10.3)	193 (12.8)	<0.001
Intermediate	497 (17.0)	321 (21.3)	
High	2,126 (72.7)	995 (65.9)	
Charlson-Deyo Comorbidity Index, n (%)			
0	2,474 (84.6)	1,270 (84.2)	0.243
1	328 (11.2)	191 (12.7)	
2	76 (2.6)	30 (2.0)	
3+	45 (1.5)	18 (1.2)	
Treatment at >1 Facility, n (%)	299 (10.2)	224 (14.8)	<0.001<0.001
Received Chemotherapy or Targeted Therapy, n (%)	174 (6.0)	24 (1.6)	
Lymph Node Surgery, n (%)			
SLNB only	1,549 (53.0)	916 (60.7)	<0.001
Regional lymph node dissection only	717 (24.5)	287 (19.0)	
SLNB and CLND in same procedure^1^	221 (7.6)	98 (6.5)	
SLNB and CLND in separate procedures	404 (13.8)	187 (12.4)	
Other or unknown^2^	32 (1.1)		
T-stage, n (%)			
T1a	311 (10.6)	138 (9.1)	0.272
T1b	613 (21.0)	314 (20.8)	
T2a	1,999 (68.4)	1,057 (70.0)	
N-stage, n (%)			
N1a	2,521 (86.2)	1,130 (74.9)	<0.001
N2a	402 (13.8)	379 (25.1)	
Ulcerated, n (%)			
Not ulcerated	2,827 (97.2)	1,413 (94.8)	<0.001
Ulcerated	82 (2.8)	78 (5.2)	
Unknown	15 (0.5)	18 (1.2)	
Mitotic Rate (mitoses/mm^2^)			
0-1	1,138 (43.6)	548 (40.7)	0.161
2-3	871 (33.4)	458 (34.0)	
≥4	602 (23.1)	340 (25.3)	
Unknown	312 (10.7)	163 (10.8)	
Follow up in months, median (IQR)	32.0 (23.4 – 40.9)	30.5 (24.2 – 38.4)	0.026
Vital status at end of follow up, n (%)			
Dead	231 (7.9)	88 (5.8)	0.011
Alive	2,692 (92.1)	1,421 (94.2)	

*Percentages presented are valid percentages (cases with missing values excluded from the denominator).

^1^Or timing of the SLNB and CLND is unable to be determined by chart review.

^2^Includes no dedicated lymph node procedure or isolated biopsy of lymph node(s) only without surgical lymph node procedure.

SD, standard deviation; IQR, interquartile range; SLNB, sentinel lymph node biopsy; CLND, completion lymph node dissection.

As the year 2017 was significant for the treatment of patients with melanoma for several reasons [MSLT-II trial results were released (28), the first PD-1 inhibitor was approved for adjuvant treatment of stage III disease (3), and the 8^th^ edition of AJCC staging criteria was released (21)], a table describing patient and facility characteristics stratified by diagnosis in or before versus after 2017 is also provided (**Supplementary Table 1**). Those diagnosed after 2017 were significantly more likely to undergo sentinel lymph node biopsy without completion lymph node dissection (81.0% vs. 39.2%, p<0.001).

### Immunotherapy utilization trends

**Figure 2:**
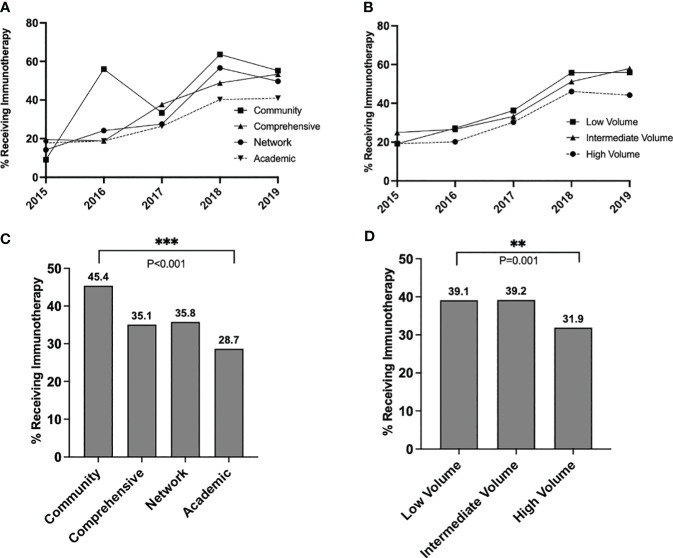
Trends in immunotherapy utilization in stage IIIA melanoma over time, stratified by **(A)** facility type and **(B)** facility volume, and overall rates of immunotherapy utilization over the entire study period, stratified by **(C)** facility type and **(D)** facility volume. P values: **<.01 and ≥.001, ***<.001.

The proportion of stage IIIA patients receiving immunotherapy increased over the study period from 20.2% in 2015 to 48.4% in 2019 (p<0.001) (**Figures 2A, B**). Throughout the study timeframe, academic centers provided less adjuvant immunotherapy to patients compared to community centers, with an overall immunotherapy percentage of 45.4% at community centers and 28.7% at academic centers (p<0.001) (**Figure 2C**). On risk-adjusted analysis, patients treated at academic centers had 52% lower odds of receiving immunotherapy than patients treated at community facilities (OR=0.48, 0.33-0.72, p<0.001) (**Supplementary Table 2**). High-volume centers also provided less immunotherapy to IIIA patients than low-volume centers (31.9% vs. 39.1%, p=0.001) (**Figure 2D**). Patients treated at high-volume centers had 31% lower risk-adjusted odds of receiving immunotherapy than patients treated at low-volume centers (Odds Ratio [OR]=0.69, 95% CI = 0.56-0.84, p<0.001) (**Supplementary Table 3**). Patients treated at comprehensive (OR=0.66, 0.45-0.96, p=0.030) or network (OR=0.67, 0.45-1.00, p=0.050) facilities were also less likely to receive immunotherapy.

Patients also had significantly lower risk-adjusted odds of receiving immunotherapy if they were older than 70 years (OR=0.55, 0.43-0.72, vs. ≤50 years old, p<0.001). Patients had significantly higher odds of receiving immunotherapy if they had N2a disease compared to N1a disease (OR=2.05, 1.74-2.41, p<0.001) or if their primary tumor was ulcerated (OR=2.09, 1.47-2.96, p<0.001). Results were similar when facility volume was used as a covariate in place of facility type (**Supplementary Table 3**).

**Figure 3:**
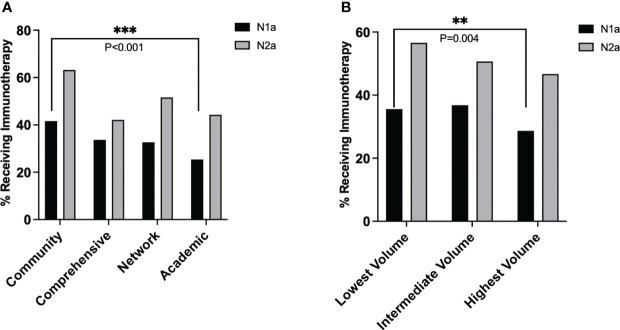
Average rate of immunotherapy utilization in stage IIIA melanoma over the study period for each nodal stage, stratified by **(A)** facility type and **(B)** facility volume. P values: **<.01 and ≥.001, ***<.001.

Further analysis revealed that facility type was associated with different rates of immunotherapy utilization amongst patients with N1a disease (overall chi-squared p<0.001), but similar rates of utilization amongst patients with N2a disease (overall p=0.157) (**Figure 3A**). In a pairwise comparison, academic centers provided significantly less immunotherapy than community centers to patients with N1a disease (25% vs. 42%, p<0.001). Similarly, immunotherapy utilization differed by volume status for N1a patients (overall p<0.001) but not N2a patients (overall p=0.206). In a pairwise comparison, high-volume centers provided less immunotherapy to N1a patients than low-volume centers (28.7% vs. 35.6%, p=0.004) (**Figure 3B**).

### Survival analysis by immunotherapy receipt, facility type, and hospital volume

**Figure 4:**
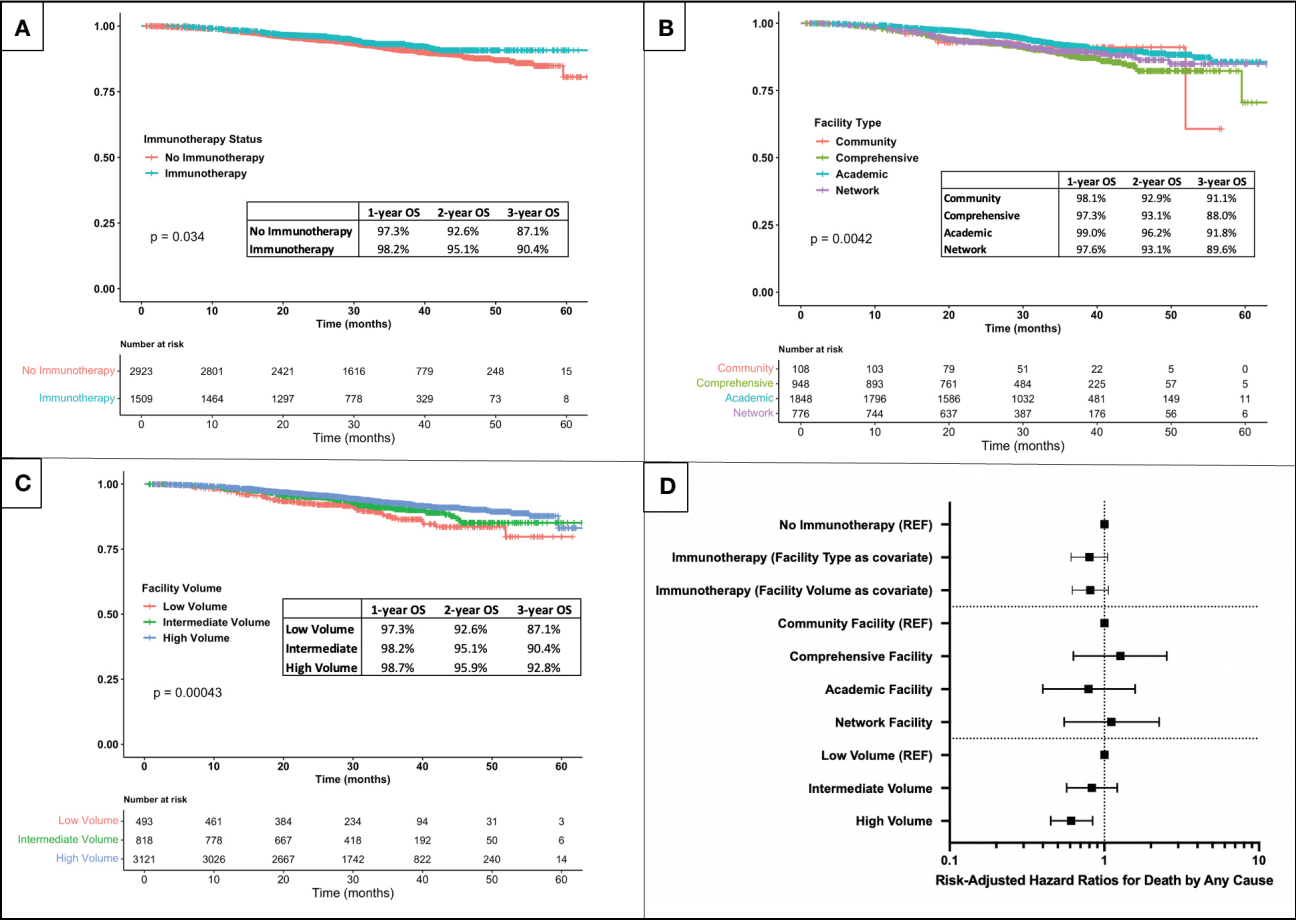
Kaplan-Meier survival analysis for patients with stage IIIA melanoma stratified by **(A)** immunotherapy receipt, **(B)** facility type, and **(C)** facility volume. **(D)** Forest plot of risk-adjusted hazard ratios for death by any cause associated with immunotherapy receipt, facility type, and facility volume (results of multivariable Cox hazards regression adjusted for age group (≤50, 51-70, >70), sex, facility location, zip code income, facility type or volume, Charlson-Deyo comorbidity index, T-stage, N-stage, ulceration status, mitotic rate, scope of lymph node surgery, and immunotherapy receipt).

Within the mean follow up time of 31.8 months, immunotherapy status was associated with overall survival on unadjusted Kaplan-Meier analysis (log-rank p=0.034), with those receiving immunotherapy living longer (**Figure 4A**). Facility type was also associated with survival (log-rank p=0.004) (**Figure 4B**), with an apparent survival advantage for those treated at academic centers. Last, facility volume was associated with unadjusted survival (log-rank p<0.001), with an apparent survival advantage for those treated at high-volume centers (**Figure 4C**).

However, in an adjusted survival analysis using the pooled results of imputed data sets, the association of immunotherapy status with overall survival lost significance (p=0.107 with facility type as a covariate and p=0.125 with facility volume as a covariate), as did the association of facility type with survival (p>0.500 for all with community centers as reference) (**Figure 4D**). The association of hospital volume with survival, however, was found to persist even after risk-adjustment: patients treated at high-volume centers had a 39% lower mortality rate as those treated at low-volume centers (HR=0.61, 0.45-0.84, p=0.002).

### Predictors of death by immunotherapy status

We next assessed association between patient and facility characteristics with death from any cause for patients that did and did not receive immunotherapy. Amongst patients who did not receive immunotherapy, the factor most protective against death was female sex (HR=0.57, 0.43-0.76, p<0.001). A factor strongly associated with an increased risk of death was Charlson-Deyo score (HR for score ≥3 = 5.32, 2.93-9.67 vs. score 0, p<0.001). Older age was also associated with mortality: compared to those ≤50, those aged 51-70 (HR=2.29, 1.44-3.65, p<0.001) and age >70 (HR=6.13, 3.83-9.81, p<0.001) experienced higher mortality. With respect to characteristics of the cancer, N2a nodal status was associated with higher mortality than N1a status (HR=1.95, 1.44-2.64, p<0.001), ulceration was associated with higher mortality (HR=3.09, 1.52-6.27, p=0.002), and mitotic rate >4 was associated with higher mortality than mitotic rate 0-1 (HR=1.84, 1.31-2.58, p<0.001) (**Supplementary Table 4**). Facility type was not associated with mortality. When using facility volume as a covariate rather than facility type, high-volume was associated with a significant decrease in risk of mortality (HR=0.63, 0.43-0.92 vs. low-volume, p=0.016).

With the exception of mitotic rate ≥4 mitoses/mm^2^, the associations of these factors with mortality in the immunotherapy cohort were similar in direction and magnitude to those for the cohort that did not receive immunotherapy (**Supplementary Table 5**). In the immunotherapy cohort, mitotic rate ≥4 mitoses/mm^2^ was not associated with greater mortality than mitotic rate 0-1 mitoses/mm^2^ (p=0.670). Given these findings, a *post-hoc* analysis was performed comparing the unadjusted overall survival of those that did versus did not receive immunotherapy, stratified by mitotic rate (**Supplementary Figure 1**). For patients with mitotic rate 0-1 or 2-3 mitoses/mm^2^, the survival curves for those receiving and not receiving immunotherapy were not different from one another (log-rank p=0.650 and p=0.300, respectively). For patients with mitotic rate ≥4, immunotherapy was associated with improved survival (p=0.047).

## Discussion

In this national assessment of the management of patients with stage IIIA melanoma from 2015-2019, immunotherapy was utilized in 34% of cases. Patients that were younger and with a higher burden of nodal disease were favored for immunotherapy treatment. We found that patients with stage IIIA melanoma treated at high-volume and academic centers had 31% and 52% lower risk-adjusted odds of receiving immunotherapy than those treated at low-volume and community centers, respectively, with no compromise in overall survival. In fact, patients treated at high-volume centers experienced a 39% lower risk-adjusted mortality compared to those treated at low-volume centers. This finding supports the conclusion that more judicious utilization of adjuvant immunotherapy in stage IIIA patients does not lead to increased death.

Differences in immunotherapy utilization by facility type have been previously reported for stage IV melanoma. Specifically, academic facilities have been found to use more immunotherapy in stage IV disease than community facilities, and outcomes are not the same: patients treated at academic facilities experience longer survival than those treated at community facilities (17, 29). It is reasonable to attribute this survival difference, at least in part, to greater immunotherapy utilization at academic centers given robust level 1 data demonstrating a significant survival advantage for immunotherapy in stage IV disease (7, 18, 19).

The fact that immunotherapy is more heavily used by academic facilities in stage IV melanoma contrasts with our finding that these same facilities use *less* immunotherapy for stage IIIA patients: it appears that patients treated at academic facilities – whether with stage IIIA disease receiving less immunotherapy or with stage IV disease receiving more immunotherapy – experience equal or better survival compared to those treated at community facilities. Although immunotherapy practices capture only part of the complex care of these patients, these findings, taken together, suggest that more restrictive immunotherapy utilization in patients with stage IIIA disease is not detrimental to patient survival compared to a more liberal prescribing practice.

This variation in immunotherapy utilization for stage IIIA disease is observed in the setting of limited data for the indication. Although immunotherapy may improve recurrence free survival in stage IIIB and IIIC disease, there are no randomized data demonstrating improved survival with immunotherapy for stage IIIA patients. The real-world data from our study also fail to demonstrate improved risk-adjusted overall survival for IIIA patients who received immunotherapy over those that did not, further reinforcing the published randomized data from smaller cohorts. Another recent real-world study of 183 patients with stage IIIA melanoma using the Flatiron database identified a trend towards longer overall survival in those receiving adjuvant nivolumab, but this trend did not reach statistical significance (30).

In light of ambiguous guidelines, institutional experience with immunotherapy and clinician experience with these agents and with immune-related adverse events (irAEs) may factor heavily into the decision to prescribe immunotherapy. Clinicians at academic and high-volume centers may be more exposed to rare and severe irAEs and therefore may be more cautious to prescribe immunotherapy for borderline indications. Academic institutions that were sites for early clinical trials in melanoma have likely been treating melanoma patients with immune checkpoint inhibitors for a longer period than community facilities and may therefore have more experience with stage-specific outcomes. This difference in experience is particularly noteworthy for a rare stage of disease like IIIA: 50% of centers in this study treated only 1-2 stage IIIA patients over the four years of the study. Academic referral centers may also be more comfortable managing distant disease and therefore more comfortable delaying immunotherapy until there is clinical evidence of metastasis.

Prominent current guidelines for the treatment of stage IIIA melanoma rely on degree of nodal involvement to determine which patients should receive immunotherapy (10–12). One important finding from this study is that academic and high-volume centers prescribed the same amount of immunotherapy as community and low-volume facilities to patients with N2a disease, but less immunotherapy to patients with N1a disease. Across all facility types and volumes, N2a nodal status was associated with an approximately 2-fold increase in odds of receiving immunotherapy. In summary, all clinicians appear to prescribe more immunotherapy to patients with higher nodal burden of disease; facilities that prescribed immunotherapy restrictively, however, were more likely to withhold immunotherapy specifically from patients with low burden of nodal disease. It should be noted that some other patient and disease factors likely used by clinicians to guide the decision to recommend adjuvant immunotherapy are not captured by the NCDB, including size of lymph node implants, functional and performance status, and patient comorbidities.

In an effort to identify predictors of response to immunotherapy within the IIIA patient population, we performed multivariable Cox proportional hazards regressions in those that received and those that did not receive immunotherapy. We found that most predictors of death were similar in both groups. For example, patients over the age of 70 were about 6 to 7 times more likely to die than those 50 or younger within the timeframe of the study, whether or not they received immunotherapy. In other words, older patients have higher mortality due to advanced age, but do not appear to experience any excess mortality from immunotherapy compared to younger patients. Mitotic rate ≥4 mitoses/mm^2^, however, was a negative prognostic factor for those who did not receive immunotherapy (HR=1.8, p<0.001) but not for those that did (HR=1.1, p=0.670). Moreover, immunotherapy appeared to be associated with longer survival for patients with mitotic rate ≥4 mitoses/mm2, but not for patients with lower mitotic rate. This may suggest that patients with high mitotic rate – a characteristic well-demonstrated to be a negative prognostic marker for melanoma (31, 32) – realize greater benefit from adjuvant immunotherapy than those with low mitotic rates. Further research to determine which stage IIIA melanoma patients will derive benefit from or be harmed by adjuvant immunotherapy is needed.

In interpreting the results of this study, it is important to consider its limitations. First, the National Cancer Database (NCDB) is retrospective in nature, and therefore this study comes with all limitations inherent to retrospective analysis, including difficulty comprehensively adjusting for confounders and sources of bias, as well as issues with differential loss to follow up and missing data. The NCDB does not report size of metastases, so we are unable to distinguish between N1a patients with <1mm lymph node disease and those with >1mm of lymph node disease. The NCDB also does not report specific immunotherapeutic agent utilized, and we are therefore unable to distinguish between treatment with high-dose interleukin-2, anti-CTLA-4 antibodies, and anti-PD-1 antibodies. In terms of outcomes, the NCDB only reports overall survival, so we are unable to comment on melanoma-specific survival or disease-free survival in this cohort. Furthermore, a consequence of evaluating only recent data in this study is that the median follow up of the cohort was less than three years, which may be too short to adequately demonstrate differences in survival for these patients, even in a large cohort.

A final limitation is the low number of patients treated at community facilities. Due to the NCDB criteria for community designation, which includes a limit on the number of cancer patients treated per year (27), it is unavoidable that the number of patients in this database treated at these facilities would be small. We attempted to mitigate the impact of this low sample size by also reporting results by hospital volume, which allowed for similar analyses with larger sample sizes. We considered the practices of community and low-volume centers to be of particular importance, as it is likely that there are a number of centers like these that treat melanoma patients whose outcomes are not captured by the NCDB, as very small centers are likely to not meet criteria for Commission on Cancer accreditation and therefore not be included in the database.

## Conclusion

From 2015-2019, patients with stage IIIA melanoma treated at high-volume or academic centers received significantly less immunotherapy than those treated at low-volume or community centers, but experienced similar overall survival. Patients with N1a disease were notably more likely to be excluded from immunotherapy at high-volume and academic centers. These findings indicate that more restrictive use of adjuvant immunotherapy in these patients may be reasonable. Patients with high mitotic rate, on the other hand, appear to benefit from adjuvant immunotherapy to a greater extent than those with low mitotic rate; greater granularity of long-term outcomes data is needed to identify the subpopulations most advantaged by this treatment. Ultimately, treatment guidelines for adjuvant immunotherapy in stage IIIA melanoma should be clarified in order to better standardize clinical practices and maximize benefit to patients.

## Data availability statement

The original contributions presented in the study are included in the article/**Supplementary Material**. Further inquiries can be directed to the corresponding author.

## Ethics statement

Ethical approval was not required for the study involving humans in accordance with the local legislation and institutional requirements. Written informed consent to participate in this study was not required from the participants or the participants’ legal guardians/next of kin in accordance with the national legislation and the institutional requirements.

## Author contributions

AF: Conceptualization, Data curation, Formal Analysis, Investigation, Methodology, Software, Visualization, Writing – original draft, Writing – review & editing. DK: Conceptualization, Data curation, Formal Analysis, Investigation, Methodology, Software, Visualization, Writing – original draft, Writing – review & editing. SK: Conceptualization, Project administration, Supervision, Visualization, Writing – review & editing. TT: Conceptualization, Visualization, Writing – review & editing. HK: Conceptualization, Visualization, Writing – review & editing. JC: Conceptualization, Visualization, Writing – review & editing. SA: Conceptualization, Visualization, Writing – review & editing. MS: Conceptualization, Visualization, Writing – review & editing. JI: Conceptualization, Data curation, Investigation, Methodology, Project administration, Supervision, Visualization, Writing – review & editing. KO: Conceptualization, Data curation, Methodology, Project administration, Supervision, Visualization, Writing – original draft, Writing – review & editing.

## Supplementary material

Supplementary Figure 1 Kaplan-Meier survival analysis for patients with stage IIIA melanoma stratified by immunotherapy status who had mitotic rate **(A)** 0 or 1 mitoses per mm^2^ **(B)** 2 or 3 mitoses per mm^2^ **(C)** 4 or more mitoses per mm^2^.

Supplementary Figure 2 Forest plot of risk-adjusted hazard ratios for death by any cause associated with different patient and tumor characteristics, with either facility type **(A)** or facility volume **(B)** as a covariate (results of multivariable Cox hazards regression adjusted for age group (≤50, 51-70, >70), sex, facility location, zip code income, facility type or volume, Charlson-Deyo comorbidity index, T-stage, N-stage, ulceration status, mitotic rate, scope of lymph node surgery, and immunotherapy receipt).
