# Supplementary figures and images for: Immunotherapy utilization in stage IIIA melanoma: less may be more
Source: Front Oncol. 2024 Feb 6;14:1336441. doi: 10.3389/fonc.2024.1336441 (PMC10876869; doi:10.3389/fonc.2024.1336441)

## Slide 1
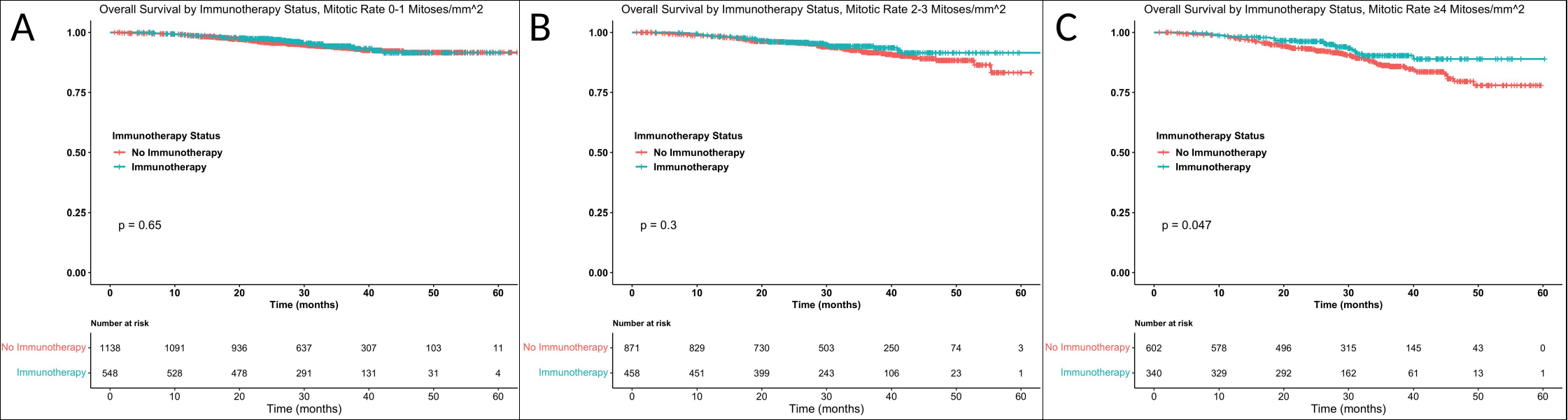

A
B
C

Supplement: Supplementary Figure 1 — Kaplan-Meier survival analysis for patients with stage IIIA melanoma stratified by immunotherapy status who had mitotic rate (A) 0 or 1 mitoses per mm2 (B) 2 or 3 mitoses per mm2 (C) 4 or more mitoses per mm2. [file Presentation_1.pptx]

## Slide 1
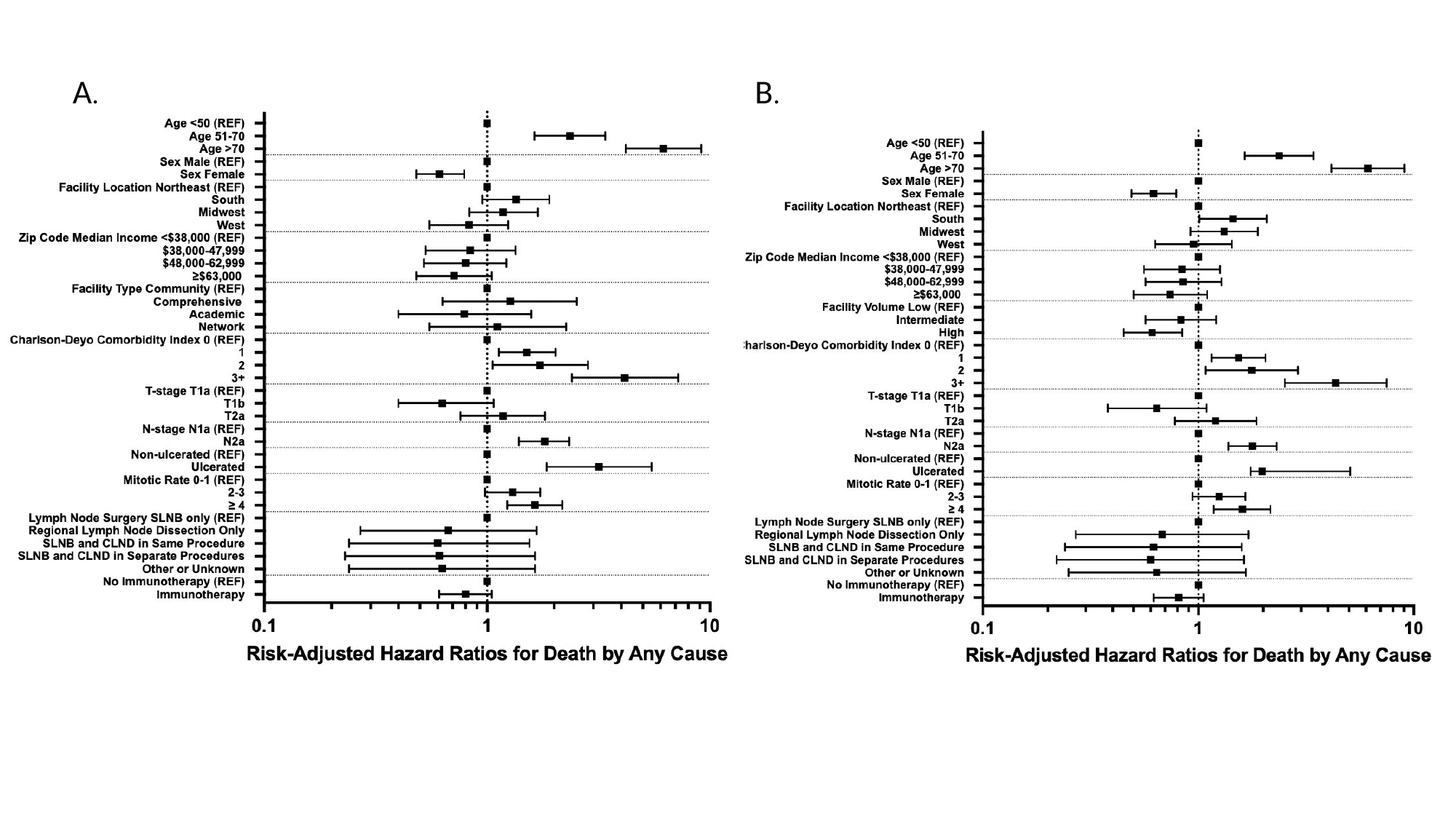

A.
B.

Supplement: Supplementary Figure 2 — Forest plot of risk-adjusted hazard ratios for death by any cause associated with different patient and tumor characteristics, with either facility type (A) or facility volume (B) as a covariate (results of multivariable Cox hazards regression adjusted for age group (≤50, 51-70, >70), sex, facility location, zip code income, facility type or volume, Charlson-Deyo comorbidity index, T-stage, N-stage, ulceration status, mitotic rate, scope of lymph node surgery, and immunotherapy receipt). [file Presentation_2.pptx]
