# Supplementary material for: Immunotherapy utilization in stage IIIA melanoma: less may be more
Source: Front Oncol. 2024 Feb 6;14:1336441. doi: 10.3389/fonc.2024.1336441 (PMC10876869; doi:10.3389/fonc.2024.1336441)
Supplement: Supplementary file 3 [file Table_1.docx]

| **Supplementary Table 1. Patient and Treatment Facility Characteristics for Stage 3A Patients Stratified by Year of Diagnosis** | | | |
| --- | --- | --- | --- |
|  | 2015-2017 | 2018-2019 | P-value |
| n (%) | 2,689 (60.7%) | 1,743 (39.3%) | - |
| Age, mean ± SD | 55.1 ± 15.3 | 56.2 ± 15.2 | 0.018 |
| Sex, n (%) |  |  |  |
| Male | 1,370 (50.9) | 983 (56.4) | <0.001 |
| Female | 1,319 (49.1) | 760 (43.6) |  |
| Race, n (%)* |  |  |  |
| White | 2,638 (98.7) | 1,711 (98.8) | 0.522 |
| Black | 7 (0.3) | 7 (0.4) |  |
| Other | 28 (1.0) | 14 (0.8) |  |
| Unknown | 16 (0.6) | 11 (0.6) |  |
| Ethnicity, n (%)* |  |  |  |
| Hispanic | 52 (2.0) | 38 (2.2) | 0.605 |
| Not Hispanic | 2,576 (98.0) | 1,684 (97.8) |  |
| Unknown | 61 (2.3) | 21 (1.2) |  |
| Facility Location, n (%)* |  |  |  |
| Northeast | 410 (18.6) | 254 (17.3) | 0.161 |
| South | 670 (30.3) | 497 (33.8) |  |
| Midwest | 677 (30.6) | 436 (29.7) |  |
| West | 453 (20.5) | 283 (19.3) |  |
| Unknown | 479 (17.8) | 273 (15.7) |  |
| Facility County, n (%)* |  |  |  |
| Metropolitan | 2,212 (84.8) | 1,412 (83.1) | 0.321 |
| Urban | 364 (13.9) | 265 (15.6) |  |
| Rural | 34 (1.3) | 23 (1.4) |  |
| Unknown | 79 (2.9) | 43 (2.5) |  |
| Zip code median income, n (%)* |  |  |  |
| < $38,000 | 218 (9.6) | 142 (9.8) | 0.770 |
| $38,000 – $47,999 | 441 (19.5) | 286 (19.8) |  |
| $48,000 – $62,999 | 635 (28.1) | 422 (29.3) |  |
| ≥$63,000 | 967 (42.8) | 592 (41.1) |  |
| Unknown | 428 (15.9) | 301 (17.3) |  |
| Insurance, n (%)* |  |  |  |
| None | 37 (1.4) | 37 (2.2) | 0.012 |
| Private | 1,718 (64.6) | 1,040 (60.5) |  |
| Medicaid | 107 (4.0) | 82 (4.8) |  |
| Medicare | 770 (29.0) | 533 (31.0) |  |
| Other government | 26 (1.0) | 28 (1.6) |  |
| Unknown | 31 (1.2) | 23 (1.3) |  |
| Facility Type, n (%)* |  |  |  |
| Community | 57 (2.6) | 51 (3.5) | 0.009 |
| Comprehensive | 591 (26.7) | 357 (24.3) |  |
| Academic | 1,130 (51.1) | 718 (48.8) |  |
| Network | 432 (19.5) | 344 (23.4) |  |
| Unknown | 479 (17.8) | 273 (15.7) |  |
| Volume, n (%) |  |  |  |
| Low | 300 (11.2) | 193 (11.1) | 0.523 |
| Intermediate | 482 (17.9) | 336 (19.3) |  |
| High | 1,907 (70.9) | 1,214 (69.7) |  |
| Charlson-Deyo Comorbidity Index, n (%) |  |  |  |
| 0 | 2,300 (85.5) | 1,444 (82.8) | 0.020 |
| 1 | 304 (11.3) | 215 (12.3) |  |
| 2 | 55 (2.0) | 51 (2.9) |  |
| 3+ | 30 (1.1) | 33 (1.9) |  |
| Treatment at >1 Facility, n (%) | 325 (12.1) | 198 (11.4) | 0.464 |
| Received Chemotherapy or Targeted Therapy, n (%) | 90 (3.4) | 108 (6.2) | <0.001 |
| Lymph Node Surgery, n (%) |  |  |  |
| SLNB only | 1,053 (39.2) | 1,412 (81.0) | <0.001 |
| Regional lymph node dissection only | 881 (32.8) | 123 (7.1) |  |
| SLNB and CLND in same procedure^1^ | 205 (7.6) | 114 (6.5) |  |
| SLNB and CLND in separate procedures | 516 (19.2) | 75 (4.3) |  |
| Other or unknown^2^ | 34 (1.3) | 19 (1.1) |  |
| T-stage, n (%) |  |  |  |
| T1a | 306 (11.4) | 143 (8.2) | <0.001 |
| T1b | 486 (18.1) | 441 (25.3) |  |
| T2a | 1,897 (70.5) | 1,159 (66.5) |  |
| N-stage, n (%) |  |  |  |
| N1a | 2,198 (81.7) | 1,453 (83.4) | 0.166 |
| N2a | 491 (18.3) | 290 (16.6) |  |
| Ulcerated, n (%) |  |  |  |
| Not ulcerated | 2,612 (97.1) | 1,628 (95.1) | <0.001 |
| Ulcerated | 77 (2.9) | 83 (4.9) |  |
| Unknown | 0 (0.0) | 32 (1.8) |  |
| Mitotic Rate (mitoses/mm^2^) |  |  |  |
| 0-1 | 1,004 (41.9) | 682 (43.7) |  |
| 2-3 | 840 (35.1) | 489 (31.3) | 0.043 |
| ≥4 | 551 (23.0) | 391 (25.0) |  |
| Unknown | 294 (10.9) | 181 (10.4) |  |
| Follow up in months, median (IQR) | 33.8 ± 12.8 | 28.7 ± 9.8 | <0.001 |
| Vital status at end of follow up, n (%) |  |  |  |
| Dead | 217 (8.1) | 102 (5.9) | 0.005 |
| Alive | 2,472 (91.9) | 1,743 (94.1) |  |
| *Percentages presented are valid percentages (cases with missing values excluded from the denominator)  ^1^Or timing of the SLNB and CLND is unable to be determined by chart review.  ^2^Includes no dedicated lymph node procedure or isolated biopsy of lymph node(s) only without surgical lymph node procedure | | | |
| Abbreviations: *SD*=standard deviation; *IQR*=interquartile range; *SLNB*=sentinel lymph node biopsy; *CLND*=completion lymph node dissection | | | |
