# Supplementary material for: Immunotherapy utilization in stage IIIA melanoma: less may be more
Source: Front Oncol. 2024 Feb 6;14:1336441. doi: 10.3389/fonc.2024.1336441 (PMC10876869; doi:10.3389/fonc.2024.1336441)
Supplement: Supplementary file 4 [file Table_2.docx]

| **Supplementary Table 2. Factors Associated with Immunotherapy Receipt (Facility Type as Covariate)** | | | |
| --- | --- | --- | --- |
|  | OR | 95% CI | P-value |
| Age Group |  |  |  |
| ≤ 50 | REF | REF | REF |
| 51-70 | 0.88 | 0.75-1.02 | 0.083 |
| **>70** | **0.55** | **0.43-0.72** | **<0.001** |
| Sex, female | 0.91 | 0.80-1.04 | 0.175 |
| Race |  |  |  |
| White | REF | REF | REF |
| Black | 1.85 | 0.61-5.63 | 0.281 |
| Other | 0.89 | 0.46-1.73 | 0.729 |
| Ethnicity, Hispanic | 1.29 | 0.82-2.01 | 0.269 |
| Facility Location |  |  |  |
| Northeast | REF | REF | REF |
| South | 1.18 | 0.95-1.46 | 0.129 |
| Midwest | 1.06 | 0.84-1.34 | 0.595 |
| West | 1.17 | 0.93-1.48 | 0.180 |
| Facility County |  |  |  |
| Metropolitan | REF | REF | REF |
| Urban | 0.91 | 0.74-1.12 | 0.388 |
| Rural | 1.07 | 0.60-1.89 | 0.820 |
| Zip code median income |  |  |  |
| < $38,000 | REF | REF | REF |
| $38,000 – $47,999 | 0.98 | 0.71-1.34 | 0.876 |
| $48,000 – $62,999 | 1.08 | 0.82-1.43 | 0.594 |
| ≥$63,000 | 0.99 | 0.73-1.34 | 0.924 |
| Insurance |  |  |  |
| None | REF | REF | REF |
| Private | 0.98 | 0.60-1.59 | 0.931 |
| Medicaid | 0.87 | 0.49-1.53 | 0.617 |
| Medicare | 0.69 | 0.41-1.16 | 0.160 |
| Other government | 1.83 | 0.88-3.79 | 0.103 |
| Facility Type |  |  |  |
| Community | REF | REF | REF |
| **Comprehensive** | **0.66** | **0.45-0.96** | **0.030** |
| **Academic** | **0.48** | **0.33-0.72** | **<0.001** |
| Network | 0.67 | 0.45-1.00 | 0.050 |
| Charlson-Deyo Comorbidity Index |  |  |  |
| 0 | REF | REF | REF |
| 1 | 1.19 | 0.98-1.46 | 0.085 |
| 2 | 0.84 | 0.54-1.31 | 0.431 |
| 3+ | 1.15 | 0.65-2.03 | 0.642 |
| T-stage |  |  |  |
| T1a | REF | REF | REF |
| T1b | 1.07 | 0.83-1.38 | 0.608 |
| T2a | 1.20 | 0.96-1.50 | 0.112 |
| N-stage |  |  |  |
| N1a | REF | REF | REF |
| **N2a** | **2.05** | **1.74-2.41** | **<0.001** |
| **Ulcerated** | **2.09** | **1.47-2.96** | **<0.001** |
| **Mitotic Rate (mitoses/mm^2^)** |  |  |  |
| **0-1** | **REF** | **REF** | **REF** |
| 2-3 | 1.09 | 0.93-1.27 | 0.291 |
| ≥4 | 1.16 | 0.97-1.37 | 0.105 |
| Abbreviations: *OR* = odds ratio; *CI* = confidence interval | | | |
