# Supplementary material for: Immunotherapy utilization in stage IIIA melanoma: less may be more
Source: Front Oncol. 2024 Feb 6;14:1336441. doi: 10.3389/fonc.2024.1336441 (PMC10876869; doi:10.3389/fonc.2024.1336441)
Supplement: Supplementary file 5 [file Table_3.docx]

| **Supplementary Table 3. Factors Associated with Immunotherapy Receipt (Volume as Covariate)** | | | |
| --- | --- | --- | --- |
|  | OR | 95% CI | P-value |
| Age Group |  |  |  |
| ≤ 50 | REF | REF | REF |
| 51-70 | 0.88 | 0.76-1.02 | 0.093 |
| **>70** | **0.56** | **0.43-0.73** | **<0.001** |
| Sex, female | 0.92 | 0.80-1.04 | 0.189 |
| Race |  |  |  |
| White | REF | REF | REF |
| Black | 1.79 | 0.59-5.45 | 0.308 |
| Other | 0.84 | 0.43-1.64 | 0.619 |
| Ethnicity, Hispanic | 1.17 | 0.75-1.82 | 0.486 |
| Facility Location |  |  |  |
| Northeast | REF | REF | REF |
| **South** | **1.27** | **1.02-1.58** | **0.031** |
| Midwest | 1.16 | 0.93-1.45 | 0.182 |
| West | 1.24 | 0.99-1.54 | 0.056 |
| Facility County |  |  |  |
| Metropolitan | REF | REF | REF |
| Urban | 0.94 | 0.77-1.15 | 0.501 |
| Rural | 1.15 | 0.62-2.11 | 0.653 |
| Zip code median income |  |  |  |
| < $38,000 | REF | REF | REF |
| $38,000 – $47,999 | 1.04 | 0.78-1.38 | 0.808 |
| $48,000 – $62,999 | 1.20 | 0.88-1.62 | 0.241 |
| ≥$63,000 | 1.09 | 0.82-1.46 | 0.547 |
| Insurance |  |  |  |
| None | REF | REF | REF |
| Private | 1.00 | 0.61-1.63 | 0.988 |
| Medicaid | 0.87 | 0.49-1.55 | 0.647 |
| Medicare | 0.69 | 0.41-1.15 | 0.155 |
| Other government | 1.91 | 0.92-3.98 | 0.083 |
| Facility Volume |  |  |  |
| Low | REF | REF | REF |
| Intermediate | 0.97 | 0.77-1.23 | 0.809 |
| **High** | **0.69** | **0.56-0.84** | **<0.001** |
| Charlson-Deyo Comorbidity Index |  |  |  |
| 0 | REF | REF | REF |
| 1 | 1.21 | 0.99-1.47 | 0.065 |
| 2 | 0.88 | 0.56-1.37 | 0.566 |
| 3+ | 1.14 | 0.64-2.02 | 0.661 |
| T-stage |  |  |  |
| T1a | REF | REF | REF |
| T1b | 1.07 | 0.83-1.38 | 0.609 |
| T2a | 1.19 | 0.95-1.49 | 0.127 |
| N-stage |  |  |  |
| N1a | REF | REF | REF |
| **N2a** | **2.05** | **1.74-2.40** | **<0.001** |
| **Mitotic Rate (mitoses/mm^2^)** |  |  |  |
| **0-1** | **REF** | **REF** | **REF** |
| **2-3** | **1.06** | **0.91-1.24** | **0.452** |
| **≥4** | **1.19** | **0.97-1.45** | **0.090** |
| **Ulcerated** | **2.09** | **1.48-2.95** | **<0.001** |
| Abbreviations: *OR* = odds ratio; *CI* = confidence interval | | | |
| *Results for volume status when analysis repeated with same covariates except volume status substituted for facility type; full results of repeat analysis available in Supplementary Table 1 | | | |
