# Supplementary material for: Immunotherapy utilization in stage IIIA melanoma: less may be more
Source: Front Oncol. 2024 Feb 6;14:1336441. doi: 10.3389/fonc.2024.1336441 (PMC10876869; doi:10.3389/fonc.2024.1336441)
Supplement: Supplementary file 6 [file Table_4.docx]

| **Supplementary Table 4. Hazard Ratios for Death by Any Cause for Patients who Did Not Receive Immunotherapy (Pooled Estimates from Imputed Datasets)** | | | |
| --- | --- | --- | --- |
|  | HR | 95% CI | P-value |
| Age Group |  |  |  |
| ≤ 50 | REF | REF | REF |
| **51-70** | **2.29** | **1.44-3.64** | **<0.001** |
| **>70** | **6.13** | **3.83-9.81** | **<0.001** |
| **Sex, female** | **0.57** | **0.43-0.76** | **<0.001** |
| Facility Location |  |  |  |
| Northeast | REF | REF | REF |
| South | 1.31 | 0.86-2.00 | 0.203 |
| Midwest | 1.28 | 0.84-1.94 | 0.254 |
| West | 0.87 | 0.53-1.41 | 0.562 |
| Zip code median income |  |  |  |
| < $38,000 | REF | REF | REF |
| $38,000 – $47,999 | 0.79 | 0.46-1.34 | 0.373 |
| $48,000 – $62,999 | 0.77 | 0.46-1.30 | 0.326 |
| ≥$63,000 | 0.67 | 0.41-1.10 | 0.109 |
| Facility Type |  |  |  |
| Community | REF | REF | REF |
| Comprehensive | 1.72 | 0.59-5.02 | 0.318 |
| Academic | 1.10 | 0.38-3.19 | 0.864 |
| Network | 1.29 | 0.44-3.83 | 0.641 |
| Charlson-Deyo Comorbidity Index |  |  |  |
| 0 | REF | REF | REF |
| **1** | **1.74** | **1.24-2.43** | **0.001** |
| 2 | 1.52 | 0.84-2.75 | 0.168 |
| **3+** | **5.32** | **2.93-9.67** | **<0.001** |
| T-stage |  |  |  |
| T1a | REF | REF | REF |
| **T1b** | **0.52** | **0.29-0.95** | **0.032** |
| T2a | 1.00 | 0.62-1.61 | 0.990 |
| N-stage |  |  |  |
| N1a | REF | REF | REF |
| **N2a** | **1.95** | **1.44-2.64** | **<0.001** |
| **Ulceration** | **3.09** | **1.52-6.27** | **0.002** |
| **Lymph Node Surgery** |  |  |  |
| SLNB only | REF | REF | REF |
| Regional lymph node dissection only | 0.59 | 0.20-1.77 | 0.348 |
| SLNB and CLND in same procedure | 0.48 | 0.16-1.46 | 0.193 |
| SLNB and CLND in separate procedures | 0.47 | 0.14-1.53 | 0.206 |
| Other or unknown | 0.50 | 0.16-1.59 | 0.242 |
| **Mitotic Rate (mitoses/mm2)** |  |  |  |
| 0-1 | REF | REF | REF |
| 2-3 | 1.37 | 0.98-1.91 | 0.062 |
| **≥4** | **1.84** | **1.31-2.58** | **<0.001** |
| Volume Status* |  |  |  |
| Low | REF | REF | REF |
| Intermediate | 0.78 | 0.49-1.24 | 0.296 |
| **High** | **0.63** | **0.43-0.92** | **0.016** |
| Abbreviations: *OR* = odds ratio; *CI* = confidence interval  *Results for volume status when analysis repeated with same covariates except volume status substituted for facility type; hazard ratios associated with other covariates were similar to those from the analysis utilizing facility type and are not presented | | | |
