# Supplementary material for: Immunotherapy utilization in stage IIIA melanoma: less may be more
Source: Front Oncol. 2024 Feb 6;14:1336441. doi: 10.3389/fonc.2024.1336441 (PMC10876869; doi:10.3389/fonc.2024.1336441)
Supplement: Supplementary file 7 [file Table_5.docx]

| **Supplementary Table 5. Hazard Ratios for Death by Any Cause for Patients who Received Immunotherapy** | | | |
| --- | --- | --- | --- |
|  | HR | 95% CI | P-value |
| Age Group |  |  |  |
| ≤ 50 | REF | REF | REF |
| **51-70** | **2.57** | **1.35-4.88** | **0.004** |
| **>70** | **7.30** | **3.32-16.02** | **<0.001** |
| Sex, female | 0.77 | 0.47-1.26 | 0.297 |
| Facility Location |  |  |  |
| Northeast | REF | REF | REF |
| South | 1.51 | 0.79-2.89 | 0.212 |
| Midwest | 1.03 | 0.52-2.02 | 0.940 |
| West | 0.78 | 0.35-1.72 | 0.533 |
| Zip code median income |  |  |  |
| < $38,000 | REF | REF | REF |
| $38,000 – $47,999 | 0.99 | 0.41-2.39 | 0.988 |
| $48,000 – $62,999 | 0.84 | 0.40-1.77 | 0.637 |
| ≥$63,000 | 0.85 | 0.41-1.79 | 0.669 |
| Facility Type |  |  |  |
| Community | REF | REF | REF |
| Comprehensive | 0.93 | 0.38-2.26 | 0.869 |
| Academic | 0.49 | 0.20-1.22 | 0.124 |
| Network | 1.09 | 0.42-2.82 | 0.859 |
| Charlson-Deyo Comorbidity Index |  |  |  |
| 0 | REF | REF | REF |
| 1 | 1.06 | 0.57-1.97 | 0.854 |
| 2 | 2.57 | 0.99-6.70 | 0.053 |
| 3+ | 1.74 | 0.37-8.30 | 0.478 |
| T-stage |  |  |  |
| T1a | REF | REF | REF |
| T1b | 0.72 | 0.23-2.26 | 0.574 |
| T2a | 1.34 | 0.53-3.40 | 0.528 |
| N-stage |  |  |  |
| N1a | REF | REF | REF |
| **N2a** | **1.73** | **1.07-2.81** | **0.026** |
| **Ulceration** | **3.77** | **1.48-9.62** | **0.006** |
| Lymph Node Surgery |  |  |  |
| SLNB only | REF | REF | REF |
| Regional lymph node dissection only | 1.04 | 0.12-8.93 | 0.968 |
| SLNB and CLND in same procedure | 1.34 | 0.53-11.9 | 0.788 |
| SLNB and CLND in separate procedures | 1.49 | 0.16-14.07 | 0.726 |
| Other or unknown | 1.52 | 0.17-13.40 | 0.704 |
| Mitotic Rate (mitoses/mm^2^) |  |  |  |
| 0-1 | REF | REF | REF |
| 2-3 | 1.12 | 0.65-1.94 | 0.667 |
| ≥4 | 1.14 | 0.61-2.13 | 0.670 |
| Volume Status* |  |  |  |
| Low | REF | REF | REF |
| Intermediate | 0.85 | 0.44-1.65 | 0.629 |
| **High** | **0.52** | **0.29-0.93** | **0.030** |
| Abbreviations: *OR* = odds ratio; *CI* = confidence interval | | | |
| *Results for volume status when analysis repeated with same covariates except volume status substituted for facility type; hazard ratios associated with other covariates were similar to those from the analysis utilizing facility type and are not presented | | | |
