# Supplementary material for: Immunotherapy utilization in stage IIIA melanoma: less may be more
Source: Front Oncol. 2024 Feb 6;14:1336441. doi: 10.3389/fonc.2024.1336441 (PMC10876869; doi:10.3389/fonc.2024.1336441)
Supplement: Supplementary file 8 [file Table_6.docx]

| **Supplementary Table 6. Risk-adjusted hazard ratios for death by any cause associated with different patient and tumor characteristics with facility type as covariate.** | | | |
| --- | --- | --- | --- |
|  | HR | 95% CI | P-value |
| Age Group |  |  |  |
| ≤ 50 | REF | REF | REF |
| 51-70 | 2.35 | 1.63-3.39 | <.001 |
| >70 | 6.19 | 4.19-9.15 | <.001 |
| Sex, female | 0.61 | 0.48-0.79 | <.001 |
| Facility Location |  |  |  |
| Northeast | REF | REF | REF |
| South | 1.35 | 0.95-1.90 | .090 |
| Midwest | 1.18 | 0.83-1.69 | .347 |
| West | 0.83 | 0.55-1.24 | .364 |
| Zip code median income |  |  |  |
| < $38,000 | REF | REF | REF |
| $38,000 – $47,999 | 0.84 | 0.53-1.34 | .451 |
| $48,000 – $62,999 | 0.80 | 0.52-1.22 | .871 |
| ≥$63,000 | 0.71 | 0.48-1.05 | .084 |
| Facility Type |  |  |  |
| Community | REF | REF | REF |
| Comprehensive | 1.27 | 0.63-2.53 | .503 |
| Academic | 0.79 | 0.40-1.58 | .511 |
| Network | 1.11 | 0.55-2.26 | .774 |
| Charlson-Deyo Comorbidity Index |  |  |  |
| 0 | REF | REF | REF |
| 1 | 1.51 | 1.13-2.03 | .005 |
| 2 | 1.73 | 1.06-2.84 | .029 |
| 3+ | 4.15 | 2.40-7.20 | <.001 |
| T-stage |  |  |  |
| T1a | REF | REF | REF |
| T1b | 0.63 | 0.40-1.07 | .090 |
| T2a | 1.18 | 0.76-1.82 | .470 |
| N-stage |  |  |  |
| N1a | REF | REF | REF |
| N2a | 1.81 | 1.39-2.34 | <.001 |
| Ulcerated | 3.18 | 1.85-5.47 | <.001 |
| Mitotic Rate (mitoses/mm^2^) |  |  |  |
| 0-1 | REF | REF | REF |
| 2-3 | 1.30 | 0.98-1.73 | .069 |
| ≥4 | 1.64 | 1.23-2.18 | <.001 |
| Lymph Node Surgery |  |  |  |
| SLNB only | REF | REF | REF |
| Regional lymph node dissection only | 0.67 | 0.27-1.67 | .386 |
| SLNB and CLND in same procedure | 0.60 | 0.24-1.55 | .293 |
| SLNB and CLND in separate procedures | 0.61 | 0.23-1.64 | .327 |
| Other or unknown | 0.63 | 0.24-1.64 | .339 |
| Immunotherapy | 0.80 | 0.61-1.05 | .107 |
| Abbreviations: HR = hazard ratio; CI = confidence interval; SLNB = sentinel lymph node biopsy; CLND = completion lymph node dissection | | | |
