# Supplementary material for: Immunotherapy utilization in stage IIIA melanoma: less may be more
Source: Front Oncol. 2024 Feb 6;14:1336441. doi: 10.3389/fonc.2024.1336441 (PMC10876869; doi:10.3389/fonc.2024.1336441)
Supplement: Supplementary file 9 [file Table_7.docx]

| **Supplementary Table 7. Risk-adjusted hazard ratios for death by any cause associated with different patient and tumor characteristics with facility volume as covariate.** | | | |
| --- | --- | --- | --- |
|  | HR | 95% CI | P-value |
| Age Group |  |  |  |
| ≤ 50 | REF | REF | REF |
| 51-70 | 2.37 | 1.64-3.42 | <.001 |
| >70 | 6.13 | 4.15-9.05 | <.001 |
| Sex, female | 0.62 | 0.49-0.79 | <.001 |
| Facility Location |  |  |  |
| Northeast | REF | REF | REF |
| South | 1.45 | 1.01-2.08 | .042 |
| Midwest | 1.32 | 0.92-1.89 | .136 |
| West | 0.95 | 0.63-1.43 | .803 |
| Zip code median income |  |  |  |
| < $38,000 | REF | REF | REF |
| $38,000 – $47,999 | 0.84 | 0.56-1.26 | .393 |
| $48,000 – $62,999 | 0.85 | 0.57-1.28 | .433 |
| ≥$63,000 | 0.74 | 0.50-1.10 | .138 |
| Facility Volume |  |  |  |
| Low | REF | REF | REF |
| Intermediate | 0.83 | 0.57-1.21 | .333 |
| High | 0.61 | 0.45-0.84 | .002 |
| Charlson-Deyo Comorbidity Index |  |  |  |
| 0 | REF | REF | REF |
| 1 | 1.54 | 1.15-2.05 | .004 |
| 2 | 1.77 | 1.08-2.90 | .024 |
| 3+ | 4.34 | 2.52-7.47 | <.001 |
| T-stage |  |  |  |
| T1a | REF | REF | REF |
| T1b | 0.64 | 0.38-1.09 | .099 |
| T2a | 1.20 | 0.78-1.86 | .402 |
| N-stage |  |  |  |
| N1a | REF | REF | REF |
| N2a | 1.78 | 1.38-2.31 | <.001 |
| Ulcerated | 2.98 | 1.75-5.08 | <.001 |
| Mitotic Rate (mitoses/mm^2^) |  |  |  |
| 0-1 | REF | REF | REF |
| 2-3 | 1.25 | 0.94-1.65 | .125 |
| ≥4 | 1.60 | 1.18-2.16 | .003 |
| Lymph Node Surgery |  |  |  |
| SLNB only | REF | REF | REF |
| Regional lymph node dissection only | 0.68 | 0.27-1.71 | .412 |
| SLNB and CLND in same procedure | 0.62 | 0.24-1.59 | .324 |
| SLNB and CLND in separate procedures | 0.60 | 0.22-1.63 | .316 |
| Other or unknown | 0.64 | 0.25-1.66 | .357 |
| Immunotherapy | 0.81 | 0.62-1.06 | .125 |
| Abbreviations: HR = hazard ratio; CI = confidence interval; SLNB = sentinel lymph node biopsy; CLND = completion lymph node dissection | | | |
